# Oral squamous cell carcinoma: metastasis, potentially associated malignant disorders, etiology and recent advancements in diagnosis

**DOI:** 10.12688/f1000research.22941.1

**Published:** 2020-04-02

**Authors:** Amr Bugshan, Imran Farooq

**Affiliations:** 1 Department of Biomedical Dental Sciences, College of Dentistry, Imam Abdulrahman Bin Faisal University, Dammam, 31441, Saudi Arabia

**Keywords:** Oral squamous cell carcinoma, Metastasis, Potentially malignant disorders, Etiological factors of OSCC, Diagnosis of OSCC

## Abstract

Oral squamous cell carcinoma (OSCC) is a commonly occurring head and neck cancer. It has a high prevalence in certain parts of the world, and is associated with a high mortality rate. In this review, we describe metastasis related to OSCC, and disorders that could lead to OSCC with common etiological factors. In addition, a brief account of the diagnosis of OSCC and role of salivary biomarkers in its early detection has also been highlighted. Google Scholar and PubMed search engines were searched with keywords including “oral squamous cell carcinoma”, “OSCC”, “oral cancer”, “potentially malignant disorders in oral cavity”, “etiological factors of OSCC”,  “diagnosis of OSCC”, and “salivary biomarkers and OSCC” to gather the literature for this review. The review concludes that OSCC has the potential for regional as well as distant metastasis, and many potentially malignant diseases can transform into OSCC with the help of various etiological factors. Diagnosis of OSCC involves traditional biopsy, but salivary biomarkers could also be utilized for early recognition.

## Introduction

**Figure 1.**
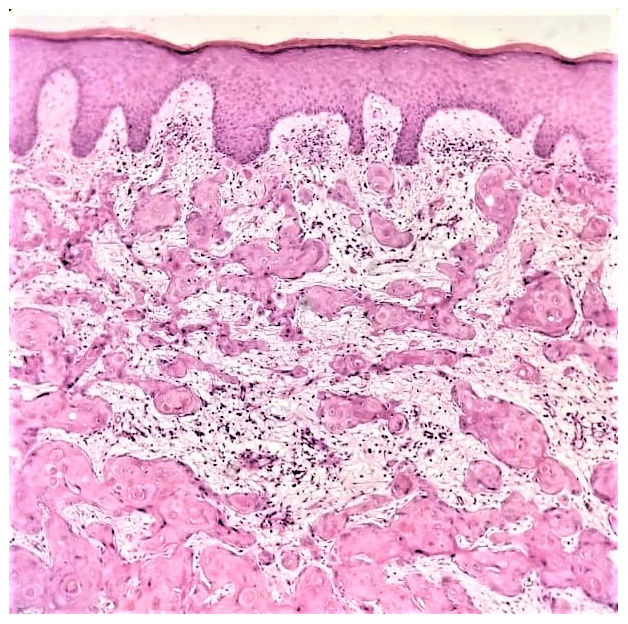
Photomicrograph showing well differentiated oral squamous carcinoma cells displaying nuclear pleomorphism, mitosis, and high number of keratin pearls. Image is courtesy of Dr. Faraz Kasti (Oral Pathology Division, College of Dentistry, Imam Abdulrahman Bin Faisal University, Dammam, Saudi Arabia). Written informed consent was obtained from the individual for publication.

One of the commonest forms of cancer is head and neck cancer ^1^. Its prevalence is different in various parts of the world; in unindustrialized countries, like India, it is the cancer most commonly diagnosed in male patients whereas in the Western world, it is responsible for 1–4% of all cancers ^2^. Lip, oral cavity, and oropharynx combined were responsible for about 4,47,751 new cancer cases with an estimated 2,28,389 deaths in 2018, which accounts for 2.4% of all cancer deaths ^3^. Among other cancers, head and neck cancer is fourteenth in terms of incidence but thirteenth in terms of mortality ^3^. The Asian continent has the highest incidence and mortality rates of oral cavity and oropharynx cancers among all other countries ^4^. More than 90% of cancer cases in head and neck region are OSCCs ( Figure 1) ^5^. OSCC develops in the oral cavity and oropharynx and can occur due to many etiological factors, but smoking and alcohol remain the most common risk factors especially in the Western world ^6^. In South Asian countries, consumption of smokeless tobacco and areca nut products are the main etiological factors associated with OSCC ^7^. Gene mutations may also cause cancer development in the pharynx and oral cavity; however, no specific gene has been identified in OSCCs ^8^. Activation of proto-oncogenes (ras, myc, EGFR) or inhibition of tumor suppressor genes (TB53, pRb, p16) by environmental factors such as smoking, irradiation, and viral infection may increase the risk of oral and oropharynx OSCC ^9^. Most of the oral and oropharynx OSCC cases occur in elderly male patients, with tonsils and tongue being the most commonly affected sites ^10^.

In this review we have briefly described metastasis related to OSCC, some disorders that could transform into OSCC with associated common etiological factors. In addition, a brief account of the diagnosis of OSCC and role of salivary biomarkers in its early detection has also been highlighted. Google Scholar and PubMed search engines were searched with keywords including “oral squamous cell carcinoma”, “OSCC”, “oral cancer”, “potentially malignant disorders in oral cavity”, “etiological factors of OSCC”, “diagnosis of OSCC”, and “salivary biomarkers and OSCC” and our search revealed 500+ results. All the articles in languages other than English and conference abstracts/presentations were excluded. Finally, 77 articles were selected for this study and included in our review. 

## Metastasis

Metastasis could be of two types; regional and/or distant metastasis, as discussed below.

### Regional metastasis

In terms of regional metastasis, nodal metastasis transpires when tumor cells at the primary site penetrate lymphatic channels and migrate to regional lymph nodes in the neck, forming a micrometastasis ^11^. Lymph node metastasis is a critical prognostic indicator for oral and oropharyngeal carcinomas ^12^. The most common site for OSCC metastasis is cervical lymph nodes, and it reduces the survival rate by 50% ^13, 14^. Cancer cells usually spread to the lymph nodes on the same side of the cancer primary site. However, contralateral or bilateral lymph nodes metastasis can rarely occur ^9^. In histopathology, tumor cells dissemination outside the lymph node capsule making the prognosis worse and reducing patient survival rate ^11^. Therefore, a thorough head and neck lymph node inspection and palpation for all first-time patients should be performed to help in early detection of cancer, which will increase the chances for successful treatment and improve prognosis ^15^.

### Distant metastasis

For distant metastasis, carcinomas require certain biological events in order to spread from the primary tumor site to an anatomically distant site. Several steps are required for cancer cells to spread from their original site to the metastatic one, as shown in the invasion-metastasis cascade ^16^. The cascade starts at the primary tumor site where the cancer cells locally breach the basement membrane to invade the surrounding extracellular matrix and connective tissue ^17^. Then, the tumor cells move to lymphatic or blood vessels and travel to distant metastatic sites. At this point, tumor cells start to extravasate from the vessels into the stroma of the metastatic site ^18^. Initially, tumor cells use the metastatic tissue microenvironment to grow and form micrometastasis. Then, tumor cells expand and colonize to start their own proliferative program and form macroscopic metastasis ^16^. The lung is the commonest site for distant metastasis for head and neck OSCC ^19^. However, metastasis to other organs, such as mediastinal nodes, liver, and bone, have been also reported ^19, 20^. Distant metastasis worsens the prognosis and reduces the chances of successful treatment ^21^. Positive regional lymph node involvement, extracapsular invasion of tumor cells, and human papilloma virus negativity are key factors that increase the risk of primary tumor cell dissemination to distant organs ^20^.

## Potentially malignant disorders (PMDs) transforming into OSCC

Early detection of cancer is a key factor for improved prognosis and increased patient survival rate. Even though the oral cavity can be easily examined and assessed by direct visual inspection, most OSCC cases are not identified early ^22^. This most likely ensues because patients do not seek dental care on a regular basis and most oral cancers in the early stages are asymptomatic ^22^. Moreover, dentists may not be aware of the different clinical presentations of OSCC and misdiagnose cancers as reactive or benign lesions ^23^. In order to help early discovery and increase the prognosis of cancers, patient awareness about regularly visiting dentists and education of dental practice staff to carefully examine the patients should be raised ^24^.

There are many PMDs in the oral cavity that have the predisposition to transform into OSCC, a few of which are discussed below in detail.

### Leukoplakia

The World Health Organization describes *“a clinical diagnosis that include any white lesion (plaque or patch) on the oral mucosa that cannot be considered clinically or pathologically as any other disease is a leukoplakia”* ^25^. In 1975, Waldron *et al*. reviewed 3,256 clinical cases defined as “leukoplakia” and found that around 80% of the cases are diagnosed microscopically as either hyperkeratosis or acanthosis ^26^. They also reported that about 17% of the cases were potentially malignant lesions (12.2% mild to moderate dysplasia and 4.5% severe dysplasia or carcinoma *in situ*) and the diagnosis of OSCC was made in about 3% of the cases that were received with the diagnosis of “leukoplakia” ^26^. Earlier, Bewley and Farwell also reported that OSCC can occur from malignant transformation of leukoplakia ^27^. Therefore, early detection of leukoplakia is key to stop their transformation into aggressive malignant OSCC, which could be hard to treat.

### Proliferative verrucous leukoplakia (PVL)

PVL is a destructive form of oral leukoplakia that clinically presents as multiple, slowly spreading white lesions with high reappearance rate and high probability of malignant transformation ^28^. A study of 47 patients diagnosed with PVL showed that around 40% of the patients developed malignant lesions (OSCC or verrucous carcinoma) during follow-up (within 2 years) ^29^. Bagán *et al*. also reported in their study that there was a high occurrence of patients with PVL developing OSCC in different sites (gingiva and palate being most common) ^30^.

### Erythroleukoplakia

Erythroleukoplakia (sometimes called speckled leukoplakia) is a mixed red and white lesion that most likely exhibits more advanced dysplastic changes in histopathological examination compared to leukoplakia ^31^. This lesion usually has irregular margins, and *Candida* colonization on these lesions is also common ^32^. The chances of speckled leukoplakia for malignant transformation is 18–47% ^33^.

### Erythroplakia

Defined as *“Any red lesion of the oral mucosa that cannot be clinically diagnosed as any other condition is called erythroplakia*” ^34^. True erythroplakia is a more alarming clinical finding compared to leukoplakia. ^9^ A retrospective study showed that 91% of 58 cases clinically observed as “erythroplakia” were diagnosed as OSCC (51%), carcinoma *in situ* or severe dysplasia (40%), or mild or moderate epithelial dysplasia (9%) ^35^. Erythroplakia and leukoplakia are usually predecessors of OSCC ^36^ and sometimes also seen adjacent to an OSCC lesion ^37^.

### Oral submucous fibrosis (OSMF)

OSMF occurs due to progressive fibrosis of the oral mucosa due to chronic use of areca nut ^38^. Patients diagnosed with OSMF are likely to develop malignant OSCC ^39^. A prospective study was carried out on 371 patients with microscopically proven diagnosis of OSCC and it was reported that around 30% of the patients (112) had a history of OSMF ^40^. However, a study carried out by Chourasia *et al*. reported an incidence of 4.2% for patients with OSMF transforming to OSCC ^39^.

### Oral lichen planus (OLP)

An immune-mediated condition that clinically may present as reticular white areas that may or may not be associated with erosive and ulcerative lesions ^41^. There is still debate whether to consider OLP as a PMDs. A previous study in which the data of 20,095 patients was assessed reported 1.1% incidence of OLP patients developing OSCC ^42^. It should be noted however, that erosive type of OLP and patients with history of smoking and alcohol use are likely to suffer from transformation of OLP to OSCC ^42, 43^. It was reported in another previous study that tumour recurrence rate of OSCC is higher in patients who had previous OLP than the patients with primary OSCC ^44^. 

## Common etiological factors of OSCC

Various etiological factors of OSCC have been reported in the literature. The most common are summarized below.

### Cigarette smoking

Cigarette smoking helps in the spread of tumors by suppressing immunity and tumor suppressor genes, most importantly p53 and PTEN ^45^. In an earlier study, al-Idrissi reviewed 65 patients with established diagnosis of head and neck OSCC and reported that the majority of these patients were men and 41.5% were smokers ^46^. In another study from China, which included 210 cases, a strong association between long term smoking and OSCC was reported ^47^. Llewelyn and Mitchell from Scotland reported in their study that out of 454 patients with confirmed oral cancer, 60% were smokers and over 95% of those lesions were OSCC ^48^.

### Alcohol consumption

A strong connection between drinking alcohol and several cancer types has been described in the literature ^49^. The synergetic effects of alcohol consumption and tobacco smoke increases the risk of OSCC by making the oral epithelium more permeable, dissolving tobacco, and promoting its penetration ^50^. However, chronic use of alcohol alone may lead to OSCC via several mechanisms, including DNA adduct formation, generation of ethanol-related reactive oxygen metabolites, and interference with the DNA-repair mechanism ^51^.

### Shammah consumption

The consumption of shammah is on the rise in many countries ^52^. It is a combination of powdered smokeless tobacco with ingredients like lime, pepper, ash, and flavoring agents, and people use it by placing it in buccal cavity till the taste penetrates ^53^. In a previous study from Jazan, Saudi Arabia, in which data from 132 patients were recorded, it was reported that the most common cancer detected was OSCC followed by thyroid cancer ^52^. Another study carried out on Yemeni shammah users concluded that there was a strong association between daily shammah usage and formation of leukoplakia (a PMD) ^54^.

### Chewing of khat

Khat is a plant that is mostly used for chewing and is a mixture of cathine and norephidrine ^55^. In a previous study, the prevalence of its consumption was found to be 23.1% among university students of Jazan, Saudi Arabia ^56^. In an earlier case report of one patient, a strong affiliation between khat chewing and growth of OSCC was reported ^57^. Sawair *et al*. also reported a strong relationship between khat chewing and development of OSCC in their study, which consisted of 649 Yemeni patients ^58^. Lukandu *et al*. reported from Kenya that chronic khat chewing could lead to abnormal epithelial thickening of oral mucosa and increased keratinization, and fibrosis ^59^. 

### Shisha (water pipe) smoking

Shisha is commonly available in restaurants, cafes, and other eatery shops in many countries and it contains a high concentration of nicotine, tar, and carbon monoxide ^60^. In water pipe smoking, smoke passes through water and there is a general idea that it is less harmful then cigarette smoking ^61^. In a recently published review, a strong association between water pipe smoking and head and neck cancers was reported ^62^. Zaid *et al*. reported in a study from Syria and Lebanon that p53 gene mutations were associated water pipe smoking in OSCC ^63^. Al-Amad carried out a study in Jordan, which revealed that 36% of their sample who had oral cancer had a habit of water pipe smoking ^64^.

## Diagnosis of OSCC

### Exfoliative cytology

Exfoliative cytology is a simple method that could prove useful in early identification of oral cancer as it is based on collection of exfoliated cells for microscopic examination ^65^. It should be noted however that cells can suffer exfoliation normally and/or in the presence of a benign or malignant disease ^66^. Therefore, the most accurate diagnosis of OSCC should only be made by biopsy.

### Biopsy

Despite the new diagnostic modalities in oral cancer detection, biopsy and histopathologic analysis remain the gold standard to diagnose OSCC ^67^. An adequate biopsy technique involves local anaesthesia administration, having sufficient width and depth of the excised tissue, correct handling of the tissue, and submission without contamination to aid an accurate definitive diagnosis ^68^. 

### Role of salivary biomarkers in detection of OSCC

The typical diagnosis of OSCC is made by clinical oral examination followed by biopsy of the suspected tissue ^69^. Unfortunately, due to this approach, most OSCC cases either go undetected (at an early stage) or are diagnosed at advanced stages ^70^. In addition, due to late diagnosis, metastasis for OSCC is very common, resulting in a 5-year survival rate of less than 50% ^71^.

Human saliva could be used for the early detection of various diseases ^72^. OSCC is very common and its early detection can improve the prognosis significantly ^73^. It has been suggested by various researchers that a specific group of protein biomarkers are increased in saliva of individuals with OSCC ^74^. Franzmann *et al*. reported CD44 as a probable biomarker of head and neck cancer whereas, Nagler *et al*. described Cyfra-21-1 and cancer antigen-25 to be potential biomarkers for oral cancer ^74, 75^. In an earlier study including 395 patients, Elashoff *et al.* stated an increase in expression of all seven transcriptomes and three proteins as possible markers for OSCC ^76^. They also reported an increase in the levels of IL-8 and subcutaneous adipose tissue in saliva exhibiting maximum levels of sensitivity and specificity to diagnose OSCC ^77^. Similarly, Arellano-Garcia *et al.* described that expression of IL8 and IL1β were increased in saliva of patients with OSCC as compared with control patients ^78^. Gleber-Netto *et al.* performed a study involving 180 patients and reported that among the proteomic markers, IL8 and IL1β concentration was greater in OSCC patients when compared with control and dysplasia patients ^79^. Awasthi performed a study that included 64 individuals with diagnosed cases of OSCC, pre-malignant conditions, and healthy controls ^80^. It was revealed from the results of that study that patients with OSCC had increased salivary levels of Cyfra-21-1, lactate dehydrogenase, and total protein concentration in comparison to other groups ^80^.

## Conclusion

Our review concludes that OSCC has the potential for regional as well as distant metastasis. Many PMDs can transform into OSCC with the help of various etiological factors. Diagnosis of OSCC involves traditional biopsy, but salivary biomarkers could also be utilized for its early diagnosis.

## Data availability

No data is associated with this article.

## References

[1] Capparuccia L Tamagnone L: Semaphorin signaling in cancer cells and in cells of the tumor microenvironment--two sides of a coin. *J Cell Sci.* 2009;122(Pt 11):1723–36. 10.1242/jcs.030197 19461072

[2] Joshi P Dutta S Chaturvedi P: Head and neck cancers in developing countries. *Rambam Maimonides Med J.* 2014;5(2):e0009. 10.5041/RMMJ.10143 24808947 PMC4011474

[3] https://gco.iarc.fr/today/data/factsheets/populations/682-saudi-arabia-fact-sheets.pdf[Accessed: 2 ^nd^March, 2020].

[4] Al-Jaber A Al-Nasser L El-Metwally A: Epidemiology of oral cancer in Arab countries. *Saudi Med J.* 2016;37(3):249–55. 10.15537/smj.2016.3.11388 26905345 PMC4800887

[5] Tandon P Dadhich A Saluja H: The prevalence of squamous cell carcinoma in different sites of oral cavity at our Rural Health Care Centre in Loni, Maharashtra - a retrospective 10-year study. *Contemp Oncol (Pozn).* 2017;21(2):178–183. 10.5114/wo.2017.68628 28947890 PMC5611509

[6] Graham S Dayal H Rohrer T: Dentition, diet, tobacco, and alcohol in the epidemiology of oral cancer. *J Natl Cancer Inst.* 1977;59(6):1611–8. 10.1093/jnci/59.6.1611 926184

[7] Muttagi SS Chaturvedi P Gaikwad R: Head and neck squamous cell carcinoma in chronic areca nut chewing Indian women: Case series and review of literature. *Indian J Med Paediatr Oncol.* 2012;33(1):32–5. 10.4103/0971-5851.96966 22754206 PMC3385276

[8] Krishna A Singh S Kumar V: Molecular concept in human oral cancer. *Natl J Maxillofac Surg.* 2015;6(1):9–15. 10.4103/0975-5950.168235 26668446 PMC4668742

[9] Neville BW Damm DD Allen CM: Oral and maxillofacial pathology. St. Louis, Mo: Saunders/Elsevier.2009 Reference Source

[10] Weatherspoon DJ Chattopadhyay A Boroumand S: Oral cavity and oropharyngeal cancer incidence trends and disparities in the United States: 2000-2010. *Cancer Epidemiol.* 2015;39(4):497–504. 10.1016/j.canep.2015.04.007 25976107 PMC4532587

[11] Sano D Myers JN: Metastasis of squamous cell carcinoma of the oral tongue. *Cancer Metastasis Rev.* 2007;26(3-4):645-62. 10.1007/s10555-007-9082-y 17768600

[12] Denis F Garaud P Manceau A: [Prognostic value of the number of involved nodes after neck dissection in oropharyngeal and oral cavity carcinoma]. *Cancer Radiother.* 2001;5(1):12–22. 10.1016/s1278-3218(00)00017-2 11236531

[13] Sharma A Kim JW Paeng JY: Clinical analysis of neck node metastasis in oral cavity cancer. *J Korean Assoc Oral Maxillofac Surg.* 2018;44(6):282–288. 10.5125/jkaoms.2018.44.6.282 30637242 PMC6327011

[14] Woolgar JA Triantafyllou A Lewis JS Jr: Prognostic biological features in neck dissection specimens. *Eur Arch Otorhinolaryngol.* 2013;270(5):1581–92. 10.1007/s00405-012-2170-9 22983222

[15] Teymoortash A Werner JA: Current advances in diagnosis and surgical treatment of lymph node metastasis in head and neck cancer. *GMS Curr Top Otorhinolaryngol Head Neck Surg.* 2012;11:Doc04. 10.3205/cto000086 23320056 PMC3544246

[16] Valastyan S Weinberg RA: Tumor metastasis: molecular insights and evolving paradigms. *Cell.* 2011;147(2):275–292. 10.1016/j.cell.2011.09.024 22000009 PMC3261217

[17] Walker C Mojares E Del Río Hernández A: Role of Extracellular Matrix in Development and Cancer Progression. *Int J Mol Sci.* 2018;19(10): pii: E3028. 10.3390/ijms19103028 30287763 PMC6213383

[18] Lambert AW Pattabiraman DR Weinberg RA: Emerging Biological Principles of Metastasis. *Cell.* 2017;168(4):670–691. 10.1016/j.cell.2016.11.037 28187288 PMC5308465

[19] Kotwall C Sako K Razack MS: Metastatic patterns in squamous cell cancer of the head and neck. *Am J Surg.* 1987;154(4):439–442. 10.1016/0002-9610(89)90020-2 3661849

[20] Duprez F Berwouts D De Neve W: Distant metastases in head and neck cancer. *Head Neck.* 2017;39(9):1733–1743. 10.1002/hed.24687 28650113

[21] Park S Han W Kim J: Risk Factors Associated with Distant Metastasis and Survival Outcomes in Breast Cancer Patients with Locoregional Recurrence. *J Breast Cancer.* 2015;18(2):160–166. 10.4048/jbc.2015.18.2.160 26155292 PMC4490265

[22] Hadzic S Gojkov-Vukelic M Pasic E: Importance of Early Detection of Potentially Malignant Lesions in the Prevention of Oral Cancer. *Mater Sociomed.* 2017;29(2):129–133. 10.5455/msm.2017.29.129-133 28883777 PMC5544450

[23] Minhas S Sajjad A Kashif M: Oral Ulcers Presentation in Systemic Diseases: An Update. *Open Access Maced J Med Sci.* 2019;7(19):3341–3347. 10.3889/oamjms.2019.689 31949540 PMC6953949

[24] Macpherson LMD: Raising awareness of oral cancer from a public and health professional perspective. *Br Dent J.* 2018;225(9):809–814. 10.1038/sj.bdj.2018.919 30412572

[25] Kramer IR Lucas RB Pindborg JJ: Definition of leukoplakia and related lesions: an aid to studies on oral precancer. *Oral Surg Oral Med Oral Pathol.* 1978;46(4):518–39. 10.1016/0030-4220(78)90383-3 280847

[26] Waldron CA Shafer WG: Leukoplakia revisited. *A clinicopathologic study 3256 oral leukoplakias*. *Cancer.* 1975;36(4):1386–1392. 10.1002/1097-0142(197510)36:4<1386::aid-cncr2820360430>3.0.co;2-7 1175136

[27] Bewley AF Farwell DG: Oral leukoplakia and oral cavity squamous cell carcinoma. *Clin Dermatol.* 2017;35(5):461–467. 10.1016/j.clindermatol.2017.06.008 28916027

[28] Thompson L: World Health Organization classification of tumours: pathology and genetics of head and neck tumours. *Ear Nose Throat J.* 2006;85(2):74. 10.1177/014556130608500201 16579185

[29] Gandolfo S Castellani R Pentenero M: Proliferative verrucous leukoplakia: a potentially malignant disorder involving periodontal sites. *J Periodontol.* 2009;80(2):274–281. 10.1902/jop.2009.080329 19186968

[30] Bagán JV Murillo J Poveda R: Proliferative verrucous leukoplakia: unusual locations of oral squamous cell carcinomas, and field cancerization as shown by the appearance of multiple OSCCs. *Oral Oncol.* 2004;40(4):440–443. 10.1016/j.oraloncology.2003.10.008 14969824

[31] Neville BW Day TA: Oral cancer and precancerous lesions. *CA Cancer J Clin.* 2002;52(4):195–215. 10.3322/canjclin.52.4.195 12139232

[32] Warnakulasuriya S: Clinical features and presentation of oral potentially malignant disorders. *Oral Surg Oral Med Oral Pathol Oral Radiol.* 2018;125(6):582–590. 10.1016/j.oooo.2018.03.011 29673799

[33] Mortazavi H Baharvand M Mehdipour M: Oral potentially malignant disorders: an overview of more than 20 entities. *J Dent Res Dent Clin Dent Prospects.* 2014;8(1):6–14. 10.5681/joddd.2014.002 25024833 PMC4091702

[34] Yardimci G Kutlubay Z Engin B: Precancerous lesions of oral mucosa. *World J Clin Cases.* 2014;2(12):866–872. 10.12998/wjcc.v2.i12.866 25516862 PMC4266835

[35] Shafer WG Waldron CA: Erythroplakia of the oral cavity. *Cancer.* 1975;36(3):1021–1028. 10.1002/1097-0142(197509)36:3<1021::aid-cncr2820360327>3.0.co;2-w 1182656

[36] Yang SW Lee YS Chang LC: Clinical characteristics of narrow-band imaging of oral erythroplakia and its correlation with pathology. *BMC Cancer.* 2015;15:406. 10.1186/s12885-015-1422-7 25975717 PMC4434519

[37] Lapthanasupkul P Poomsawat S Punyasingh J: A clinicopathologic study of oral leukoplakia and erythroplakia in a Thai population. *Quintessence Int.* 2007;38(8):e448–55. 10.1186/s12885-015-1422-7 17823667

[38] Passi D Bhanot P Kacker D: Oral submucous fibrosis: Newer proposed classification with critical updates in pathogenesis and management strategies. *Natl J Maxillofac Surg.* 2017;8(2):89–94. 10.4103/njms.NJMS_32_17 29386809 PMC5773997

[39] Chourasia NR Borle RM Vastani A: Concomitant Association of Oral Submucous Fibrosis and Oral Squamous Cell Carcinoma and Incidence of Malignant Transformation of Oral Submucous Fibrosis in a Population of Central India: A Retrospective Study. *J Maxillofac Oral Surg.* 2015;14(4):902–906. 10.1007/s12663-015-0760-y 26604461 PMC4648770

[40] Chaturvedi P Vaishampayan SS Nair S: Oral squamous cell carcinoma arising in background of oral submucous fibrosis: a clinicopathologically distinct disease. *Head Neck.* 2013;35(10):1404–1409. 10.1002/hed.23143 22972608

[41] Said-Al-Naief N Rosebush MS Lynch D: Clinical-pathological conference: case 2. *Head Neck Pathol.* 2010;4(3):221–225. 10.1007/s12105-010-0192-4 20676833 PMC2923319

[42] Aghbari SMH Abushouk AI Attia A: Malignant transformation of oral lichen planus and oral lichenoid lesions: A meta-analysis of 20095 patient data. *Oral Oncol.* 2017;68:92–102. 10.1016/j.oraloncology.2017.03.012 28438300

[43] Mignogna MD Lo Muzio L Lo Russo L: Clinical guidelines in early detection of oral squamous cell carcinoma arising in oral lichen planus: a 5-year experience. *Oral Oncol.* 2001;37(3):262–267. 10.1016/s1368-8375(00)00096-8 11287280

[44] Muñoz AA Haddad RI Woo SB: Behavior of oral squamous cell carcinoma in subjects with prior lichen planus. *Otolaryngol Head Neck Surg.* 2007;136(3):401–404. 10.1016/j.otohns.2006.09.023 17321867

[45] Gandini S Botteri E Iodice S: Tobacco smoking and cancer: a meta-analysis. *Int J Cancer.* 2008;122(1):155–164. 10.1002/ijc.23033 17893872

[46] al-Idrissi HY: Head and neck cancer in Saudi Arabia: retrospective analysis of 65 patients. *J Int Med Res.* 1990;18(6):515–519. 10.1177/030006059001800610 2292333

[47] Wang X Xu J Wang L: The role of cigarette smoking and alcohol consumption in the differentiation of oral squamous cell carcinoma for the males in China. *J Cancer Res Ther.* 2015;11(1):141–145. 10.4103/0973-1482.137981 25879352

[48] Llewelyn J Mitchell R: Smoking, alcohol and oral cancer in south east Scotland: a 10-year experience. *Br J Oral Maxillofac Surg.* 1994;32(3):146–152. 10.1016/0266-4356(94)90098-1 8068584

[49] Seitz HK Becker P: Alcohol metabolism and cancer risk. *Alcohol Res Health.* 2007;30(1):38–47. 17718399 PMC3860434

[50] Feller L Chandran R Khammissa RA: Alcohol and oral squamous cell carcinoma. *SADJ.* 2013;68(4):176–180. 23971298

[51] Liu Y Chen H Sun Z: Molecular mechanisms of ethanol-associated oro-esophageal squamous cell carcinoma. *Cancer Lett.* 2015;361(2):164–173. 10.1016/j.canlet.2015.03.006 25766659 PMC4765374

[52] Alharbi F: Incidence of head and neck cancers in Jazan province, Saudi Arabia. *Saudi J Otorhinolaryngol Head Neck Surg.* 2017;19(2):47–50. Reference Source

[53] Quadri MF Alharbi F Bajonaid AM: Oral squamous cell carcinoma and associated risk factors in Jazan, Saudi Arabia: a hospital based case control study. *Asian Pac J Cancer Prev.* 2015;16(10):4335–4338. 10.7314/apjcp.2015.16.10.4335 26028095

[54] Scheifele C Nassar A Reichart PA: Prevalence of oral cancer and potentially malignant lesions among shammah users in Yemen. *Oral Oncol.* 2007;43(1):42–50. 10.1016/j.oraloncology.2005.12.028 16759897

[55] Al-Hebshi NN Skaug N: Khat (Catha edulis)-an updated review. *Add Biol.* 2005;10(4):299–307. 10.1080/13556210500353020 16318950

[56] Alsanosy RM Mahfouz MS Gaffar AM: Khat chewing among students of higher education in Jazan region, Saudi Arabia: prevalence, pattern, and related factors. *Biomed Res Int.* 2013;2013:487232. 10.1155/2013/487232 23878809 PMC3708399

[57] Fasanmade A Kwok E Newman L: Oral squamous cell carcinoma associated with khat chewing. *Oral Surg Oral Med Oral Pathol Oral Radiol Endod.* 2007;104(1):e53–e55. 10.1016/j.tripleo.2007.01.010 17482851

[58] Sawair FA Al-Mutwakel A Al-Eryani K: High relative frequency of oral squamous cell carcinoma in Yemen: qat and tobacco chewing as its aetiological background. *Int J Environ Health Res.* 2007;17(3):185–195. 10.1080/09603120701254813 17479382

[59] Lukandu OM Koech LS Kiarie PN: Oral Lesions Induced by Chronic Khat Use Consist Essentially of Thickened Hyperkeratinized Epithelium. *Int J Dent.* 2015;2015:104812. 10.1155/2015/104812 26491446 PMC4600501

[60] Shafagoj YA Mohammed FI: Levels of maximum end-expiratory carbon monoxide and certain cardiovascular parameters following hubble-bubble smoking. *Saudi Med J.* 2002;23(8):953–8. 12235470

[61] Al Ghobain M Ahmed A Abdrabalnabi Z: Prevalence of and attitudes to waterpipe smoking among Saudi Arabian physicians. *East Mediterr Health J.* 2018;24(3):277–282. 10.26719/2018.24.3.277 29908023

[62] Patil S Awan KH Arakeri G: The relationship of "shisha" (water pipe) smoking to the risk of head and neck cancer. *J Oral Pathol Med.* 2019;48(4):278–283. 10.1111/jop.12823 30604900

[63] Zaid K Azar-Maalouf E Barakat C: p53 Overexpression in Oral Mucosa in Relation to Shisha Smoking in Syria and Lebanon. *Asian Pac J Cancer Prev.* 2018;19(7):1879–1882. 10.22034/APJCP.2018.19.7.1879 30049200 PMC6165666

[64] Al-Amad SH Awad MA Nimri O: Oral cancer in young Jordanians: potential association with frequency of narghile smoking. *Oral Surg Oral Med Oral Pathol Oral Radiol.* 2014;118(5):560–565. 10.1016/j.oooo.2014.08.002 25442492

[65] Goregen M Akgul HM Gundogdu C: The cytomorphological analysis of buccal mucosa cells in smokers. *Turk J Med Sci.* 2011;41(2):205–10. 10.3906/sag-1005-851

[66] Salih MA Bushra MO El Nabi AH: Comparison between exfoliative cytology and histopathology in detecting oral squamous cell carcinoma. *Saudi J Oral Sci.* 2017;4:46–50. 10.4103/1658-6816.200143

[67] Badvi JA Kulsoom J Ujjan IU: Recent techniques for diagnosis of oral squamous cell carcinoma. *EC Microbiology.* 2017;5(5):165–168. Reference Source

[68] Masthan KM Sankari SL Babu NA: How to help the oral pathologist in making an accurate diagnosis. *J Clin Diagn Res.* 2013;7(1):181–184. 10.7860/JCDR/2012/4967.2703 23450041 PMC3576784

[69] Fuller C Camilon R Nguyen S: Adjunctive diagnostic techniques for oral lesions of unknown malignant potential: Systematic review with meta-analysis. *Head Neck.* 2015;37(5):755–762. 10.1002/hed.23667 24596227

[70] Mascitti M Orsini G Tosco V: An Overview on Current Non-invasive Diagnostic Devices in Oral Oncology. *Front Physiol.* 2018;9:1510. 10.3389/fphys.2018.01510 30410451 PMC6209963

[71] Cristaldi M Mauceri R Di Fede O: Salivary Biomarkers for Oral Squamous Cell Carcinoma Diagnosis and Follow-Up: Current Status and Perspectives. *Front Physiol.* 2019;10:1476. 10.3389/fphys.2019.01476 31920689 PMC6914830

[72] Javaid MA Ahmed AS Durand R: Saliva as a diagnostic tool for oral and systemic diseases. *J Oral Biol Craniofac Res.* 2016;6(1):66–75. 10.1016/j.jobcr.2015.08.006 26937373 PMC4756071

[73] Sujir N Ahmed J Pai K: Challenges in Early Diagnosis of Oral Cancer: Cases Series. *Acta Stomatol Croat.* 2019;53(2):174–180. 10.15644/asc53/2/10 31341326 PMC6604559

[74] Franzmann EJ Reategui EP Carraway KL: Salivary soluble CD44: a potential molecular marker for head and neck cancer. *Cancer Epidemiol Biomarkers Prev.* 2005;14(3):735–739. 10.1158/1055-9965.EPI-04-0546 15767360

[75] Nagler R Bahar G Shpitzer T: Concomitant analysis of salivary tumor markers--a new diagnostic tool for oral cancer. *Clin Cancer Res.* 2006;12(13):3979–3984. 10.1158/1078-0432.CCR-05-2412 16818695

[76] Elashoff D Zhou H Reiss J: Prevalidation of salivary biomarkers for oral cancer detection. *Cancer Epidemiol Biomarkers Prev.* 2012;21(4):664–672. 10.1158/1055-9965.EPI-11-1093 22301830 PMC3319329

[77] Hoffmann TK Sonkoly E Homey B: Aberrant cytokine expression in serum of patients with adenoid cystic carcinoma and squamous cell carcinoma of the head and neck. *Head Neck.* 2007;29(5):472–478. 10.1002/hed.20533 17111427

[78] Arellano-Garcia ME Hu S Wang J: Multiplexed immunobead-based assay for detection of oral cancer protein biomarkers in saliva. *Oral Dis.* 2008;14(8):705–712. 10.1111/j.1601-0825.2008.01488.x 19193200 PMC2675698

[79] Gleber-Netto FO Yakob M Li F: Salivary Biomarkers for Detection of Oral Squamous Cell Carcinoma in a Taiwanese Population. *Clin Cancer Res.* 2016;22(13):3340–3347. 10.1158/1078-0432.CCR-15-1761 26847061 PMC4930722

[80] Awasthi N: Role of salivary biomarkers in early detection of oral squamous cell carcinoma. *Indian J Pathol Microbiol.* 2017;60(4):464–8. 10.4103/IJPM.IJPM_140_16 29323056

