# Supplementary material for: Oral squamous cell carcinoma: metastasis, potentially associated malignant disorders, etiology and recent advancements in diagnosis
Source: F1000Res. 2020 Apr 2;9:229. [Version 1] doi: 10.12688/f1000research.22941.1 (PMC7194458; doi:10.12688/f1000research.22941.1)
Supplement: Peer review history

# Peer review: Reviewer response for version 1

**DOI:** 10.5256/f1000research.25327.r61966

**Published:** 2020-04-14

**Authors:** Ali Leghari

**Affiliations:** 1 Department of Community Dentistry, Baqai Dental College, Baqai Medical University, Karachi, Pakistan

The article is well written and I would like to accept it. I do have the following suggestions to improve its overall impact.

1. In figure 1: Addition of a clinical picture showing OSCC would benefit the article.
2. It would be better if PVL is mentioned as a distinct form of oral leukoplakia and not as a destructive form.
3. It would be better if areca nut as a risk factor of OSCC also mentioned separately in heading “Common etiological factor of OSCC”. 

Is the review written in accessible language?

Yes

Are all factual statements correct and adequately supported by citations?

Yes

Are the conclusions drawn appropriate in the context of the current research literature?

Yes

Is the topic of the review discussed comprehensively in the context of the current literature?

Yes

Reviewer Expertise:

NA

I confirm that I have read this submission and believe that I have an appropriate level of expertise to confirm that it is of an acceptable scientific standard.

# Peer review: Reviewer response for version 1

**DOI:** 10.5256/f1000research.25327.r61967

**Published:** 2020-04-14

**Authors:** Fatima Qadir, Ambreen Rehman

**Affiliations:** 1 Queen Mary University of London, London, UK; 2 Centre of clinical and diagnostic oral sciences, Institute of Dentistry, Barts & The London School of Medicine and Dentistry, Queen Mary University of London, London, UK

The review on Oral squamous cell carcinoma is a well written comprehensive article. It summarises the aetiology, metastasis and current diagnostic methods of OSCC. It also sheds light on possible use of salivary proteins to aid diagnosis. All the information provided is scientifically valid.

 I would like the author to review the following minor suggested changes:

1. Recheck reference 1, I have not found any content related to the incidence of OSCC or Head and neck cancer.
2. Human Papilloma Virus (HPV) is one of the leading cause of OSCC. It should be included in the aetiology.

Is the review written in accessible language?

Yes

Are all factual statements correct and adequately supported by citations?

Yes

Are the conclusions drawn appropriate in the context of the current research literature?

Yes

Is the topic of the review discussed comprehensively in the context of the current literature?

Yes

Reviewer Expertise:

Head and neck oral squamous cell carcinoma, micro-vesicles, Exosomes, oral cancer diagnostics.

We confirm that we have read this submission and believe that we have an appropriate level of expertise to confirm that it is of an acceptable scientific standard.

# Peer review: Reviewer response for version 1

**DOI:** 10.5256/f1000research.25327.r61965

**Published:** 2020-04-09

**Authors:** Faisal Aljofi

**Affiliations:** 1 Department of Oncology and Diagnostic Sciences, School of Dentistry, University of Maryland, Baltimore, MD, USA

1. In etiology section: it has been mentioned that there is a various etiological factors that cause OSCC, most of the articles that have been cited are based on epidemiological statistics. So I think it is better to specify that these are based on epidemiological statistics. 
2. It has been mentioned that early detection of cancer using certain techniques like salivary bio markers will lead to successful prognosis and treatment, are there any studies showing that there is improved prognosis after early detection? If not then this would be a good way to mention this as a suggestion and future trend to establish studies to show the essential role of early diagnosis. 
3. Suggestions to add diagnostic criteria for early detection of OSCC.

Is the review written in accessible language?

Yes

Are all factual statements correct and adequately supported by citations?

Yes

Are the conclusions drawn appropriate in the context of the current research literature?

Yes

Is the topic of the review discussed comprehensively in the context of the current literature?

Yes

Reviewer Expertise:

NA

I confirm that I have read this submission and believe that I have an appropriate level of expertise to confirm that it is of an acceptable scientific standard.
